# Photodynamic therapy dosimetry using multiexcitation multiemission wavelength: toward real-time prediction of treatment outcome

**DOI:** 10.1117/1.JBO.25.6.063812

**Published:** 2020-04-03

**Authors:** Monirehalsadat Mousavi, Lilian Tan Moriyama, Clovis Grecco, Marcelo Saito Nogueira, Katarina Svanberg, Cristina Kurachi, Stefan Andersson-Engels

**Affiliations:** a Lund University, Department of Physics, Biophotonics Group, Lund, Sweden; b University of São Paulo, São Carlos Institute of Physics, Optics Group, São Carlos/SP, Brazil; c Tyndall National Institute, IPIC, Biophotonics@Tyndall, Lee Maltings, Cork, Ireland; d University College Cork, Department of Physics, Cork, Ireland

**Keywords:** biomedical optics, fluorescence, photodynamic therapy, optical properties, dosimetry, *in vivo* measurement, fiber-optic probe

## Abstract

Evaluating the optical properties of biological tissues is needed to achieve accurate dosimetry during photodynamic therapy (PDT). Currently, accurate assessment of the photosensitizer (PS) concentration by fluorescence measurements during PDT is typically hindered by the lack of information about tissue optical properties. In the present work, a hand-held fiber-optic probe instrument monitoring fluorescence and reflectance is used for assessing blood volume, reduced scattering coefficient, and PS concentration facilitating accurate dosimetry for PDT. System validation was carried out on tissue phantoms using nonlinear least squares support machine regression analysis. It showed a high correlation coefficient (>0.99) in the prediction of the PS concentration upon a large variety of phantom optical properties. *In vivo* measurements were conducted in a PDT chlorine e6 dose escalating trial involving 36 male Swiss mice with Ehrlich solid tumors in which fluences of 5, 15, and $40\;J\;{\mathrm{cm}}^{-2}$ were delivered at two fluence rates (100 and $40\;\mathrm{mW}\;{\mathrm{cm}}^{-2}$). Remarkably, quantitative measurement of fluorophore concentration was achieved in the *in vivo* experiment. Diffuse reflectance spectroscopy (DRS) system was also used to independently measure the physiological properties of the target tissues for result comparisons. Then, blood volume and scattering coefficient measured by the fiber-optic probe system were compared with the corresponding result measured by DRS and showed agreement. Additionally, tumor hemoglobin oxygen saturation was measured using the DRS system. Overall, the system is capable of assessing the implicit photodynamic dose to predict the PDT outcome.

## 1 Introduction

Advances in optical techniques have provided noninvasive diagnostics and effective means to improve clinical outcomes.^1^^–^^3^ Fiber-optic probes have been increasingly utilized to provide a minimally invasive approach in tissue characterization for various biomedical applications, such as cancer diagnostics, surgical guidance, and treatment response monitoring.^4^^–^^7^ Photodynamic therapy (PDT) in conjunction with fiber-optic probing is a promising modality to achieve optimal therapeutic efficiency for cancer treatment.^8^^–^^10^ PDT is a viable minimally invasive treatment, involving the administration of photosensitizer (PS), incubation time to allow the adequate accumulation of the drug in the tumor, and activation by light of appropriate wavelength. This process results in the generation of highly active oxygen radicals that cause tumor necrosis, apoptosis, and autophagy.^11^^–^^13^

Although PDT has proven to be a promising modality for a variety of malignant and premalignant conditions, customized dosimetry is in high demand and development of such advanced techniques is under clinical evaluation.^14^^–^^16^ Most PDT clinical protocols utilize the explicit dosimetry based on the light and PS parameters as well as the incubation time and treatment interval to create a dose model. These parameters are dynamically interdependent and additional factors may influence the PDT outcome. One considerable issue is to know how the light is transported within the tissue to enable optimization of the treatment results. It is well known that a primary challenge of many diagnostics as well as treatment methods stem from spatial and temporal alterations in light attenuation caused by inter- and intrapatient variation of the optical properties in the tissue. Also, due to individual tumor and patient variations, including intra- and interindividual optical property distributions and PS accumulation, a single and efficient PDT irradiation protocol is many times considered unfeasible.

Evaluation of PS concentration during PDT treatment is an important implicit dosimetry metric that incorporates PS uptake and photobleaching during the treatment. Several investigators have proposed ways to monitor PS levels during PDT. Depending on the type of optical instrumentation used for illumination and detection, fiber-optic probes that measure both fluorescence and diffuse reflectance signals enable the assessment of endogenous and exogenous fluorophores as well as photobleaching products. Fiber-optic probes are placed in direct contact with the tissue to mitigate the influence of scattering and absorption on the measured fluorescence signal. Such compact fluorescence spectrometer was developed by Nadeau et al.^17^ to monitor the photobleaching of aminolevulinic acid (ALA)-induced protoporphyrin IX (PpIX) and to quantify the photobleaching rates in the skin of healthy volunteers. The instrument employs blue and red light sources and shows that the photobleaching rate for a fixed excitation fiber is wavelength dependent. It also allows to quantify the PpIX fluorescence photobleaching rates for the two excitation wavelengths. However, as their probe is designed to interrogate fluorescence with a single fiber, it is not suitable for sampling PS from beneath the superficial tissue. In another study,^18^ a dynamic model was developed to describe the photobleaching process of the involved chromophores in the probed tissue. This model can be used to achieve a photobleaching-insensitive method to improve the fluorophore quantification. Kanick et al.^16^ have reported on a spectroscopic dosimeter that combines two-channel excitation fluorescence system (blue and red excitation wavelength, respectively) with a broadband reflectance spectroscopic correction to quantify PpIX signals originating from different depths. In a similar approach, Valdes et al.^19^ have combined fluorescence and reflectance spectroscopy for *in vivo* quantification of cancer biomarkers in low- and high-grade glioma surgery based on multivariate analysis. The optical fiber was an intraoperative tool that allowed the neurosurgeon to rapidly switch between blue and white light. This group has also proposed an analytical model to extract the quantitative fluorescence signal from PpIX in guided resection surgery of brain tumors.^20^

There are a number of imaging modalities of the interest for PDT dosimetry,^14^^,^^21^ however, only few of them are able to quantitatively assess photosenstitizer concentration and tissue optical properties.^22^ Variation in the absorption and scattering coefficients may be mistaken for variations in fluorophore concentration. Therefore, an instrument that robustly assesses absorption, scattering, and fluorescence concentration could improve the irradiation dosimetry planning and treatment outcome. In this study, a fiber-optic probe system described previously in Ref. 23 was used to quantify parameters of importance for PDT dosimetry. The multichannel dosimeter used in this study is based on fluorescence and reflectance spectroscopy, and consists of five excitation wavelengths to assess fluorophore concentration and reduced scattering and absorption coefficients of the probed tissue. Multiexcitation sources have the potential to improve the accuracy of quantitative fluorescence measurements in highly distorted media while compensating for absorption caused by endogenous chromophores and blood content. The system performance was validated by tissue phantom measurements followed by multivariate analysis. An *in vivo* study on an Ehrlich solid tumor model was conducted. The objective of the present work is to evaluate the feasibility of using a hand-held fiber-optic probe to characterize optical properties of biological tissue during PDT treatment. Real-time dosimetry is important for accurate prediction of the PDT outcome and the presented system has the capability to measure all relevant PDT parameters in a short time and independent of the ambient light conditions. The evaluation of these parameters allows to retrieve the dosimetric value when the physical properties of the tissue are unknown. In a previous study conducted with this system, insensitivity to ambient light was demonstrated as well as the capability of detection of very low concentrations of the fluorophore in range of nM.^23^

## 2 Materials and Methods

### 2.1 Animals and Tumor Induction

Thirty-six male Swiss mice weighting around 30 g were subcutaneously inoculated with a suspension of $2\times 10^{6}$ Ehrlich carcinoma cells on their backs.^24^ The tumor grew for 10 days until the tumor reached about 10 mm in diameter. The animals were then divided into two experimental groups and treated using two fluence rates 40 and $100\;\mathrm{mW}\;{\mathrm{cm}}^{-2}$ of a diode laser at 660 nm. The treatment procedure was carried out according to the protocol presented in Table 1. The study was approved by the Ethics Committee for the Use of Animals of the São Carlos Institute of Physics of the University of São Paulo in compliance with the laws on experimental animals. The study was performed at the São Carlos Institute of Physics of the University of São Paulo.

**Table 1:** The protocol of the study. Thirty-six mice were involved in the *in vivo* study, each with one tumor on their back. Mice were categorized into two groups of fluence rate (100 and $40\;\mathrm{mW}\;{\mathrm{cm}}^{-2}$) and three subgroups of dose (5, 15, and $40\;J\;{\mathrm{cm}}^{-2}$).

Fluence rate ($\mathrm{mW}\;{\mathrm{cm}}^{-2}$) at 660 nm	Dose ($J\;{\mathrm{cm}}^{-2}$)	Number of mice (all = 36)
100 (Group I) (3 subgroups)	5	18
	5, 15	12
	5, 15, 40	6
40 (Group II) (3 subgroups)	5	18
	5, 15	12
	5, 15, 40	6

### 2.2 Procedures for *In Vivo* Measurements

A stock solution of Chlorine e6 (synthesized at the Department of Chemistry of the Federal University of São Carlos)^25^ was prepared using 1 mg of the PS in powder diluted in 1 μl dimethyl sulfoxide (DMSO; LabSynth^®^ Ltda, Diadema, SP, Brazil) and distilled water was added up to 1 ml, obtaining a 1 mg ml^−1^ solution. The animals were anesthetized using inhalation anesthesia (isoflurane, 2% in oxygen) and the hair of an area around the tumor about 2 cm in diameter was removed, then a dose of $2\;\mathrm{mg}\;{\mathrm{kg}}^{-1}$ of chlorine e6 was injected via the tail vein with a 4 h drug-light interval (DLI) according to the protocol. During the DLI, the animals were kept in their cages protected from ambient light. For the PDT procedure, the mice were anesthetized again and a mask made of aluminum foil covered with white bandage tape with a 5 mm in diameter hole was used to delimitate the treatment area. As a PDT light source, a 660-nm diode laser was used (QuantumTech, São Carlos, SP, Brazil) and the light was delivered through an optical fiber tip with a uniform circular illumination spot (FD Frontal Light Distributor, Medlight, Switzerland). The fluence was checked with a power meter, keeping the fiber at a fixed distance from the power meter. The animals were euthanized directly after completion of PDT irradiation and subsequent dosimetry measurements. The group of 36 animals was divided into two experimental groups, as presented in Table 1, categorized based on two different PDT fluence rates. Each of these groups was divided into three subgroups for three different total light doses. All mice in each main group (18 mice) received $5\;J\;{\mathrm{cm}}^{-2}$ dose. From then on, 12 of them received an additive of $10\;J\;{\mathrm{cm}}^{-2}$ and thereafter, 6 mice received an additive of $25\;J\;{\mathrm{cm}}^{-2}$ dose (Table 1). The two monitoring optical systems described below were used to measure the reflection and fluorescence spectra before and after PDT treatment using an optical fiber probe in gentle contact with the surface of the tumors. An initial measurement series was performed on all mice before the PS injection. The PDT light source illuminated through a hole in an aluminum foil placed on the mice skin. The consecutive optical measurements were performed at five sites on each tumor by repositioning the fiber between measurements. The measured optical properties from the five sites in each tumor were averaged. The same measurements were then repeated at the same locations immediately after each stage of the PDT light delivery.

### 2.3 System Description

Two systems described in detail below were used in this study. The first is a probe dosimeter system with five excitation sources and five detection units. This multiexcitation multiemission system (MEME) has been primarily developed for precise tumor delineation in brain surgery and is capable of suppressing ambient light as well as accurate assessment of both fluorophore concentration and tissue optical properties of tissue samples. The system has been described in detail elsewhere.^23^ The second is a diffuse reflectance spectroscopy (DRS) system. This system is, in this study, used for *in vivo* validation of the diffuse reflectance results obtained by the MEME probe dosimeter system.

#### 2.3.1 Multiexcitation and multiemission system

**Fig. 1:**
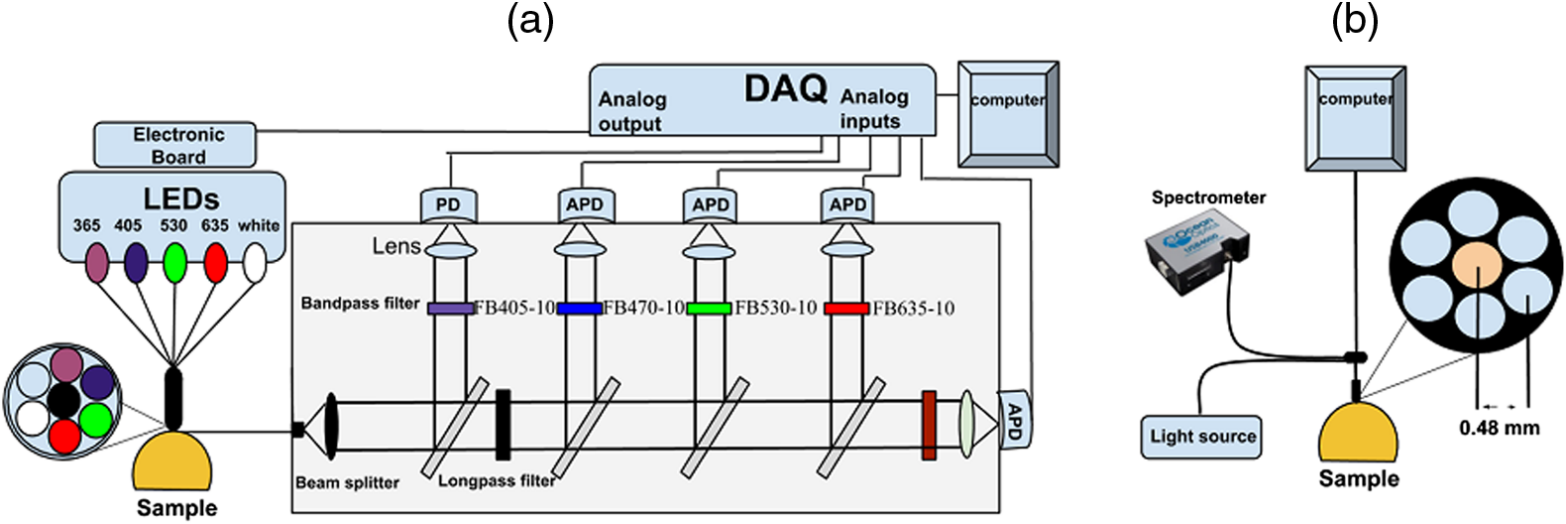
Schematic diagram of (a) MEME and (b) the optical setup for DRS.

The schematic diagram of the MEME system is shown in Fig. 1(a). The light source assembly consists of five light-emitting diodes (LEDs); four different wavelengths: 365, 405, 530, 635 nm, and one white-light LED (450 to 700 nm). The two LEDs emitting light at the shortest wavelengths were chosen to excite tissue autofluorescence. Additionally, the 405-nm LED provides a strong excitation signal for chlorine e6 fluorescence. The main tissue absorber interfering with the PDT process is blood. This is primarily absorbing the violet and green LED radiation. The present system is designed to be used in other studies employing PpIX as a photosensitizer, thus, in this study, the 635-nm LED channel is used for diffuse reflectance measurements only. All LEDs were multiplexed sequentially by rapid switching between channels, as driven by a transistor–transistor logic pulsing at 777 Hz. One measurement period contains six pulse sequences. The first five sequences were used to take measurements once, each LED is sequentially switched on while the ambient light level is acquired during the sixth pulse. A hand-held fiber-optical probe delivers light to the sample by means of six illumination fibers in a circular arrangement and with a single collection fiber at the center, each with a core diameter of 750  μm, cladding distance of <15  μm, a numerical aperture of 0.5, and a length of 4 m. The center-to-center distance between two adjacent cores is the same as the fiber cladding diameter. During the measurements, the probe tip was held perpendicularly in gently contact (with minimum pressure on the tissue) with the tissue surface. The collected light from the sample was guided to the detection unit and split into five different light paths by means of four dichroic beam splitters. The first detector is a silicon photodiode (PD; Edmund Optics, 53378) that was used to detect diffusely reflected UV and violet light from the probed tissue. Four avalanche photodiodes (APD; Hamamatsu, S9075) were utilized to collect fluorescence light arising from tissue endogenous substances, exogenous chlorine e6 fluorescence, and also reflectance from the red, green, and white LED.

#### 2.3.2 Pulse generation and data analysis

**Fig. 2:**
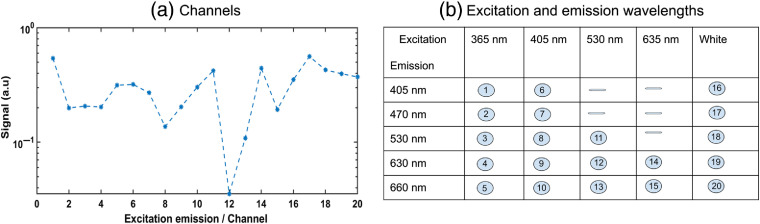
(a) Typical optical data collected by the MEME system are shown for each pair of excitation and emission wavelengths. (b) These pairs are shown in the table. The plot on the left shows the optical data collected by the five photodetectors when LEDs are on sequentially.

A digital clock output of a 16-bit DAQ board (National Instrument Corp., NIUSB-6351) was used to modulate the light source and record the signal voltages in the PD and APDs. As described above, light intensity modulation was employed, first, to measure the signal generated for the five LEDs independently and second, to enable measurements in strong ambient light conditions by means of precise background light subtraction. A full measurement cycle including all 20 measurement channels is presented in Fig. 2(a). The used excitation–emission combination defined in Fig. 2(b) is such a cycle. For a typical measurement case, the pulse generation, DAQ, real-time control, and initial data analysis are performed by a custom-made LabVIEW application. A MATLAB toolbox for least-squares support vector machines (LS-SVMs) was employed for multivariate regression and analysis.

#### 2.3.3 Statistics

LS-SVM algorithms are a set of supervised learning methods that analyze data and recognize patterns to be used in regression and classification analysis. SVM constructs a hyperplane or set of hyperplanes to transform the observations in the input space to a high-dimensional feature space with a maximal distance between support vectors of different classes in the margins. LS-SVMs are a class of kernel-based learning methods, where the kernel function separates data with increasing dimensionality. For LS-SVMs, a Gaussian radial basis function kernel was used. In this work, LS-SVM regression with twofold cross validation was used to train and evaluate the model. Parameters evaluated are the PS concentration, the reduced scattering coefficient, and the absorption coefficient. These parameters are evaluated using 56 data sample (112 data sample in total). Six randomly selected training and evaluation sets were tested, yielding very similar results, indicating that the model is not overtrained. The result of the regression is graphically shown in box plots to display five-number summary: the minimum, the maximum, the sample median, and the first and third quartiles (Figs. 5–7).

#### 2.3.4 System validation

Phantom experiments were carried out to construct a data set used for evaluation of the MEME system performance. A set of tissue-like phantoms was prepared by mixing water, Intralipid^®^ (Sigma-Aldrich, $200\;\mathrm{mg}\;{\mathrm{ml}}^{-1}$), and bovine blood (purchased from a local supermarket). Chlorine e6 stock solution was prepared by dissolving 0.3 g chlorine e6 powder in 2  μl DMSO (Merck, Darmstadt, Germany) and 80  μl distilled water to make a chlorine e6 concentration of 5  μM. In total, 112 phantoms were prepared containing different amounts of intralipid (2%, 4%, 6%, and 8%), bovine blood (1%, 2%, 3%, and 4%), and chlorine e6 (0 to 5  μM). These concentrations were chosen to have a good correspondence to real biological tissue. The phantom with the highest fluorophore concentration (5  μM) was placed in a cylindrical glass container and stirred for 4 h prior and during the measurements. The lower concentration was subsequently prepared by diluting the initial liquid tissue phantom without any fluorophore. The reduced scattering coefficient of each phantom was estimated by the Flock et al.^26^ relation:

$$\mu_{S}^{\prime }(\lambda )=C[0.58(\lambda /1\;\mu m)-0.1]0.32{\left(\lambda /1\;\mu m\right)}^{-2.4},$$ (1)

where C denotes the concentration of 20% intralipid in the phantom and λ refers to the light wavelength in μm.

### 2.4 Diffuse Reflectance Spectroscopy

#### 2.4.1 System description

Diffuse reflectance spectra were collected using an optical measurement system shown in Fig. 1(b), consisting of a halogen lamp (HL-2000, Ocean Optics) used as a light source, and a fiber-optic based spectrometer (USB4000, Ocean Optics) used to acquire diffuse reflectance spectra from 400 to 650 nm. The fiber-optic probe [Avantes, Reflection Probe (FCR-7IR400-2-ME)] consists of six surrounding collection fibers and one illumination fiber located at the center. The numerical apertures of the illumination fiber and the detection fibers are 0.22. The core diameters of the fibers are 400  μm while cladding is 480  μm. The distance between the centers of the illumination and the collection fibers is 0.48 mm.

#### 2.4.2 Data acquisition

The spectra were obtained from the same location on samples, as specified for the MEME system. A spectrally flat white reflectance standard (Spectralon, Labsphere, Inc., SRS-99-010) was used as a reflectance standard and all spectra were normalized to the calibration spectrum. The background signal was recorded and subtracted from all measurements including the reference spectra to correct for dark current and stray light.

#### 2.4.3 Inverse Monte Carlo spectral fitting

The spectral fitting was performed by using an inverse Monte Carlo algorithm. This algorithm allows the retrieval of optical properties (scattering and absorption) by relating them to reflectance values of a database generated by using forward Monte Carlo simulations (performed by using a CUDAMCML code^27^). The simulations assume a given geometric configuration of the illumination and collection fibers as well as the refractive index of the fibers and tissue. Details about the calculation of the blood volume fraction are described elsewhere.^28^

## 3 Results

### 3.1 MEME System Calibration

**Fig. 3:**
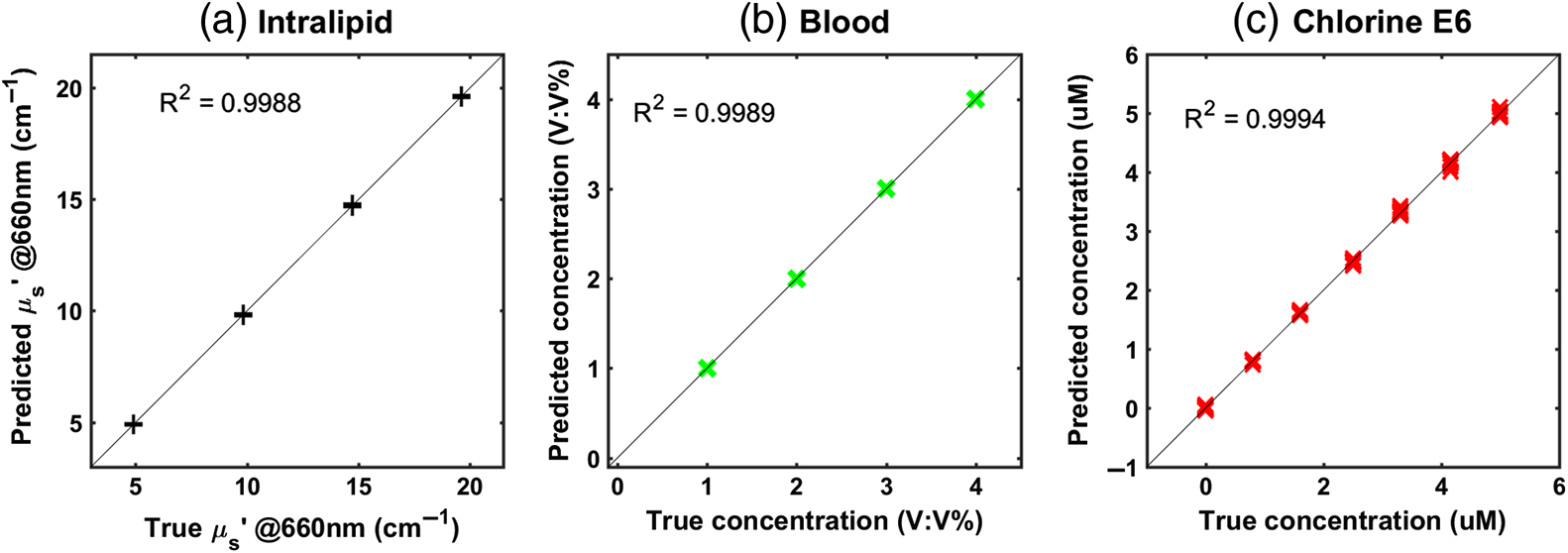
System validation plot by means of intralipid phantoms with various optical properties using a twofold LS-SVM regression method (112 samples). Each graph shows the prediction values versus true values for (a) reduced scattering coefficient (set by intralipid concentration), (b) blood as absorption coefficient (set by blood volume fraction), and (c) chlorine e6 concentration (set by the PS fluorescence) to quantify PS concentration. $R^{2}$ is the mean correlation coefficient (six repetition) between the true parameter and model response for training. The coefficient of variation value for predicted data is for each concentration below 3% for intralipid, below 1% for blood, and below 3% for chlorine e6.

The calibration responses, based on an LS-SVM regression model to predict reduced scattering coefficient ($\mu_{s}^{\prime }$), blood volume, and PS concentration, are shown in Fig. 3. The performance of each experiment was evaluated by using a twofold cross-validation methodology: the phantom dataset was split into one subset for training and another for testing. The solid line indicates supreme prediction and the black, red, and green dots represent the predicted points. The results obtained show calibration curves with correlation coefficient of ∼0.999. It indicates an excellent performance of the MEME system for the estimation of a large range of optical properties and low fluorophore concentrations in the μM range.

### 3.2 Spectral Fitting

**Fig. 4:**
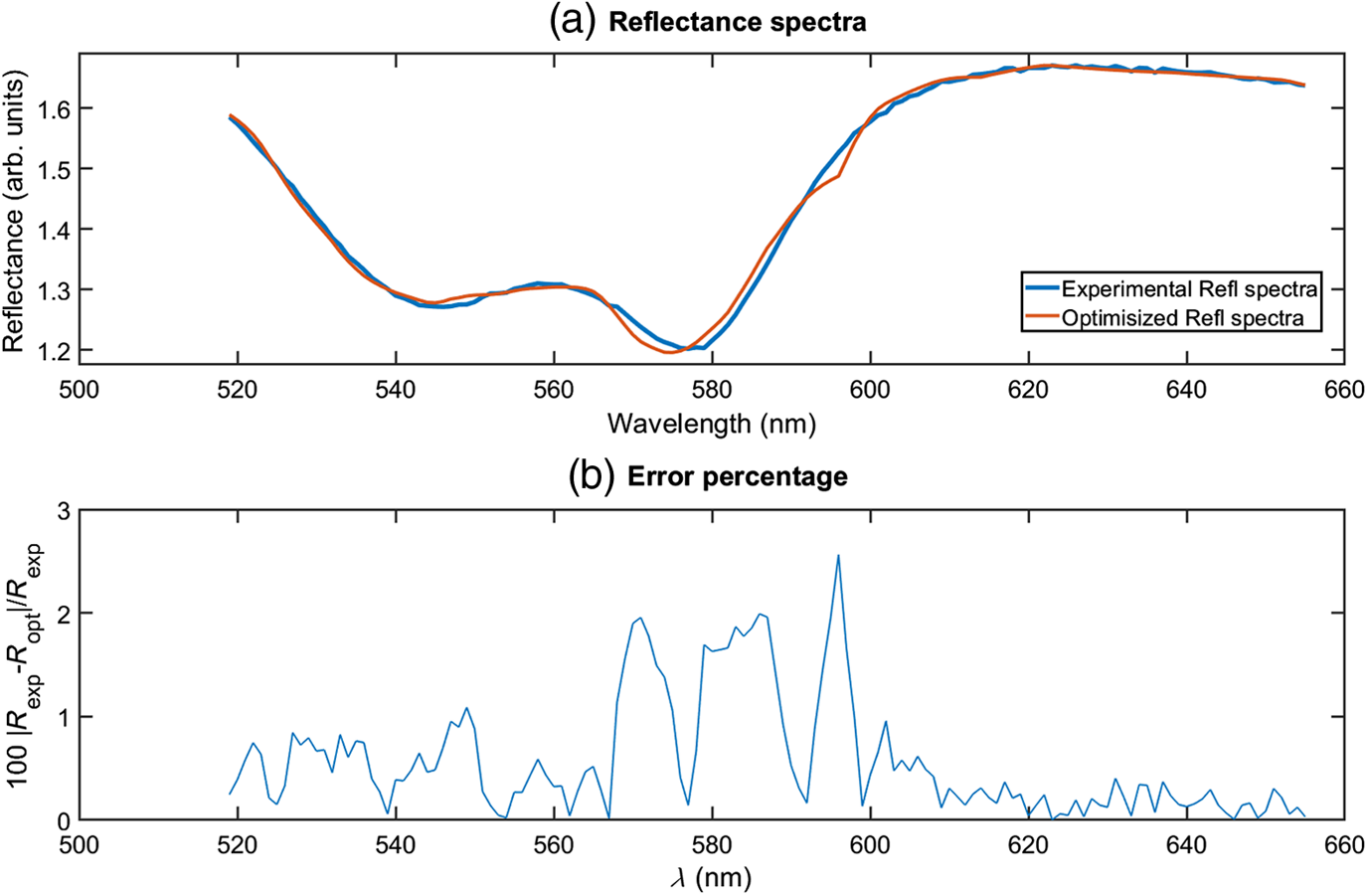
(a) DRS spectrum and inverse Monte Carlo fitted spectrum and (b) error percentage for the fitting algorithm.

Figure 4 shows comparison between an example of an experimental spectrum and the inverse Monte Carlo spectral fitting. The blue line indicates measured spectra and the corresponding model fit is shown in the red line. The chromophore parameters for the spectral fitting algorithms are hemoglobin, deoxyhemoglobin, lipid, water, and bilirubin. In addition to scattering and absorption parameters, the tissue oxygenation was also calculated by using the output values of the fitting algorithm. Error percentage is calculated as the difference between two spectra divided by the experimental reflectance is below 3.0%

### 3.3 *In Vivo* Quantification

**Fig. 5:**
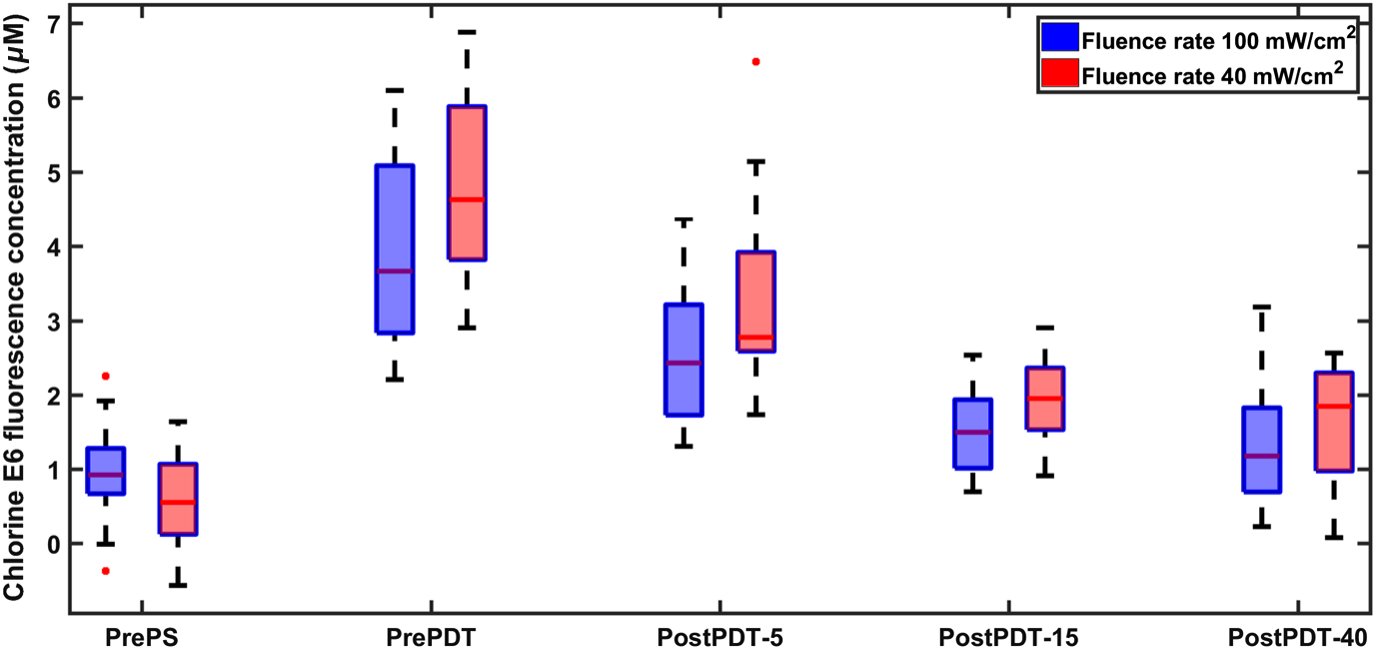
Chlorine e6 level in tumor tissues evaluated by the MEME system before drug injection, pre-PDT, and post-PDT. PrePS stands for before PS injection, PrePDT for 4 h after injection and before PDT treatment, and post-PDT-5, 15, or 40 for the three different treatment procedures after light delivery. First three box plots on the left represent measurements on 36 animals divided into two groups of PDT fluence rate (18 animals in each group). The two last box plots show 24 treated animals with $15\;J\;{\mathrm{cm}}^{-2}$ (12 animals in each group) and 12 treated animals with $40\;J\;{\mathrm{cm}}^{-2}$ (6 animals in each group). All data are based on training set obtained from tissue phantoms.

The SVM model created from phantom validation measurements is applied to the mice data for predicting the optical properties and fluorophore concentration in tumors. Since one of the main aims of this experiment was to quantitatively measure the PS accumulation in the tumors, the preinjection tissue autofluorescence, pre-PDT, and post-PDT chlorine e6 fluorescence signals were analyzed to extract the chlorine e6 concentration (Fig. 5). A low initial presence of red fluorescence signal is observed before PS injection. Accumulation of the PS is obvious after injection with three times increase in signal (p value <0.01) from the initial red fluorescence intensity for group I and seven times increase (p value <0.01) for group II. The PS intensity decreased for both groups after $5\;J\;{\mathrm{cm}}^{-2}$ treatment step. There is no remarkable difference after $15\;J\;{\mathrm{cm}}^{-2}$ delivered dose.

**Fig. 6:**
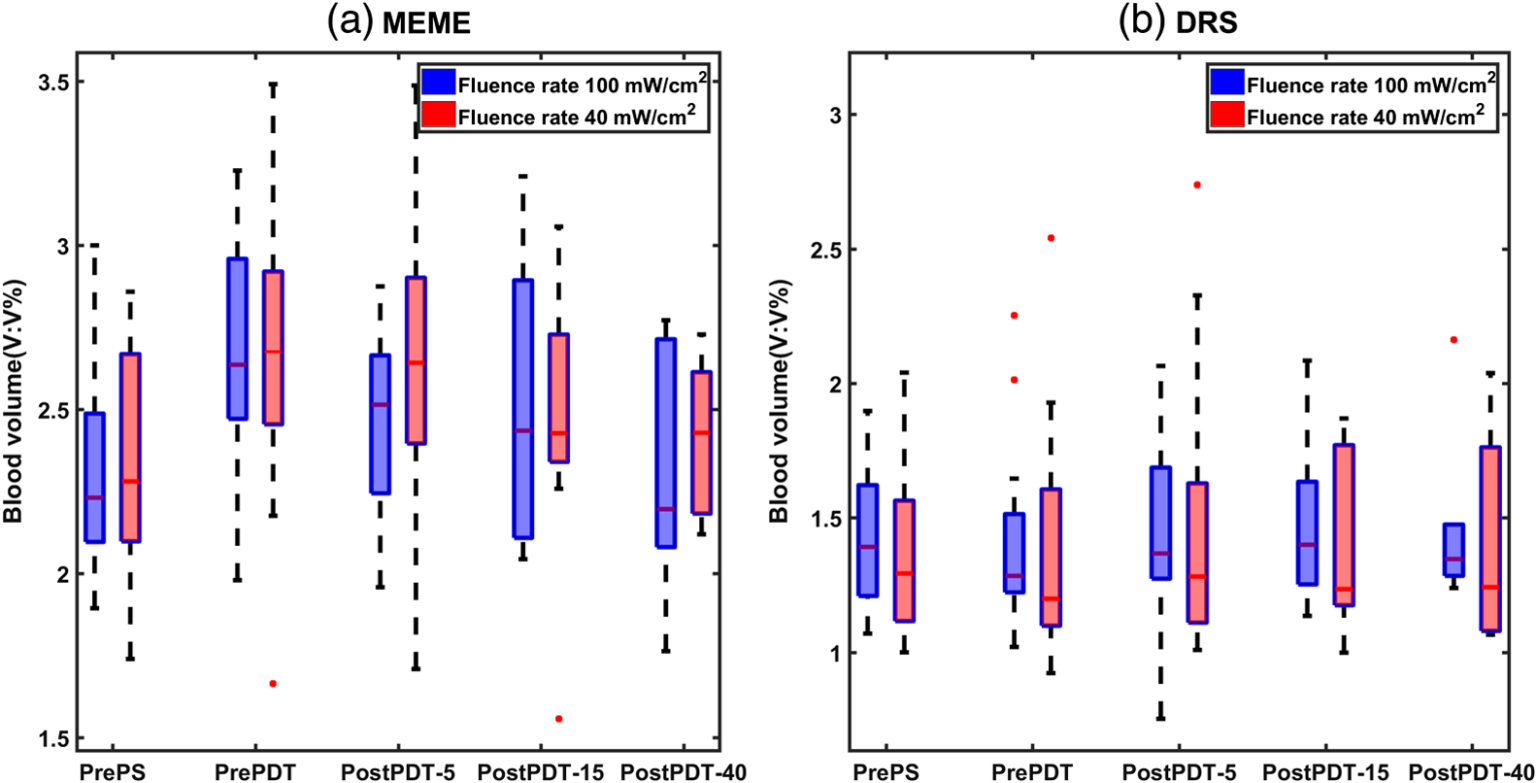
Blood volume variation of tumor tissues evaluated by the MEME and DRS system before drug injection, pre-PDT and post-PDT. PrePS stands for before PS injection, PrePDT for 4 h after injection and before PDT treatment, and postPDT-5, 15, or 40 for the three different treatment procedures after light delivery. First three box plots on the left represent measurements on 36 animals divided into two groups of PDT fluence rate (18 animals in each group). The two last box plots show 24 treated animals with $15\;J\;{\mathrm{cm}}^{-2}$ (12 animals in each group) and 12 treated animals with $40\;J\;{\mathrm{cm}}^{-2}$ (6 animals in each group).

**Fig. 7:**
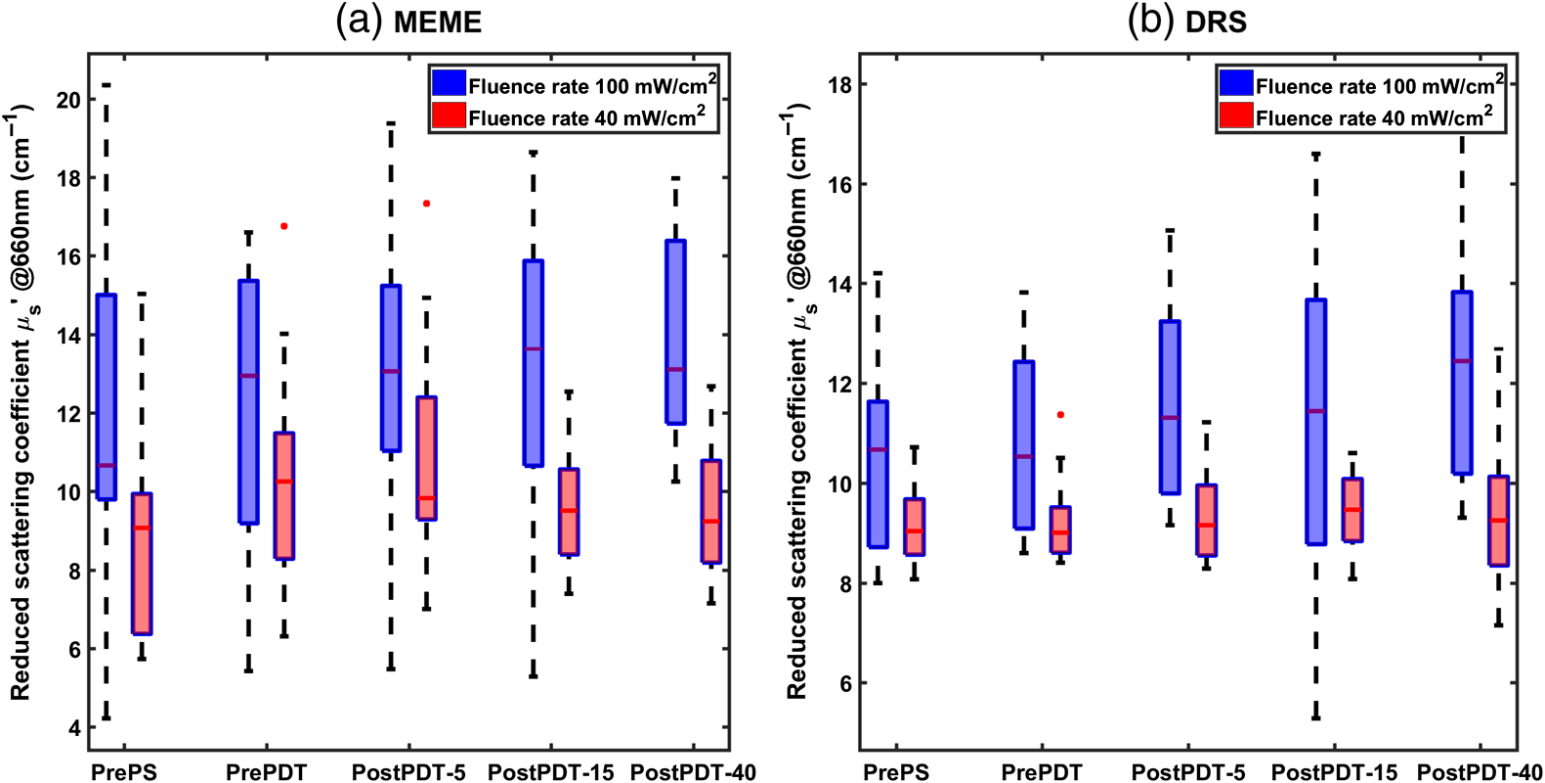
Reduced scattering coefficients of tumor tissues evaluated by MEME and DRS before drug injection, pre-PDT, and post-PDT. PrePS stands for before PS injection, PrePDT for 4 h after injection, and before PDT treatment, and postPDT-5, 15, or 40 for the three different treatment procedures after light delivery. First three box plots on the left represent measurements on 36 animals divided into two groups of PDT fluence rate (18 animals in each group). The two last box plots show 24 treated animals with $15\;J\;{\mathrm{cm}}^{-2}$ (12 animals in each group) and 12 treated animals with $40\;J\;{\mathrm{cm}}^{-2}$ (6 animals in each group).

Figures 6 and 7 illustrate the blood volume and the reduced scattering coefficient variation at different treatment procedures. Data before the PS injection were assessed to investigate whether the tumor physiology properties changed due to the drug injection. For the MEME system, the blood volume fraction is evaluated at the two fluence rates of $100\;\mathrm{mW}\;{\mathrm{cm}}^{-2}$ (group I) and $40\;\mathrm{mW}\;{\mathrm{cm}}^{-2}$ (group II) with median values of 2.2% and 2.3%, respectively, before injection. Median relative value of the blood content at group I is 1.2% after injection and 1.1%, 1.1%, 1.0%, respectively, after treatment stages with 5, 15, and $40\;J\;{\mathrm{cm}}^{-2}$. For group II, median relative blood content is 1.2% after injection, and 1.2%, 1.0%, 1.0% after the three stages of treatment. For the DRS system, the median relative change in the blood absorption for group I is 1.0% for all four measurement procedures relative to the blood absorption value before PS injection. For group II, the median relative blood volume is 1.0%.

For the MEME system, the reduced scattering coefficients before PS injection were evaluated as $10.7\;{\mathrm{cm}}^{-2}$ for group I and $9.0\;{\mathrm{cm}}^{-2}$ for group II. Comparison of the reduced scattering coefficient at two different light doses shows significantly lower value at $40\;\mathrm{mW}\;{\mathrm{cm}}^{-2}$ before PDT and during subsequent treatment. All measurement procedures demonstrated relative median values of around 1 for optical properties variation. The optical properties did not change substantially before and after PDT treatment. The reduced scattering coefficient had rather high standard deviations compared with the blood volume fraction variations. The reduced scattering coefficient exhibited a slight tendency to increase during treatment.

Blood oxygenation changes in tissue were determined by fitting the diffuse reflectance spectra based on the concentrations of oxyhemoglobin and deoxyhemoglobin. A typical fitted result for the optical parameters is depicted in Fig. 4.

**Fig. 8:**
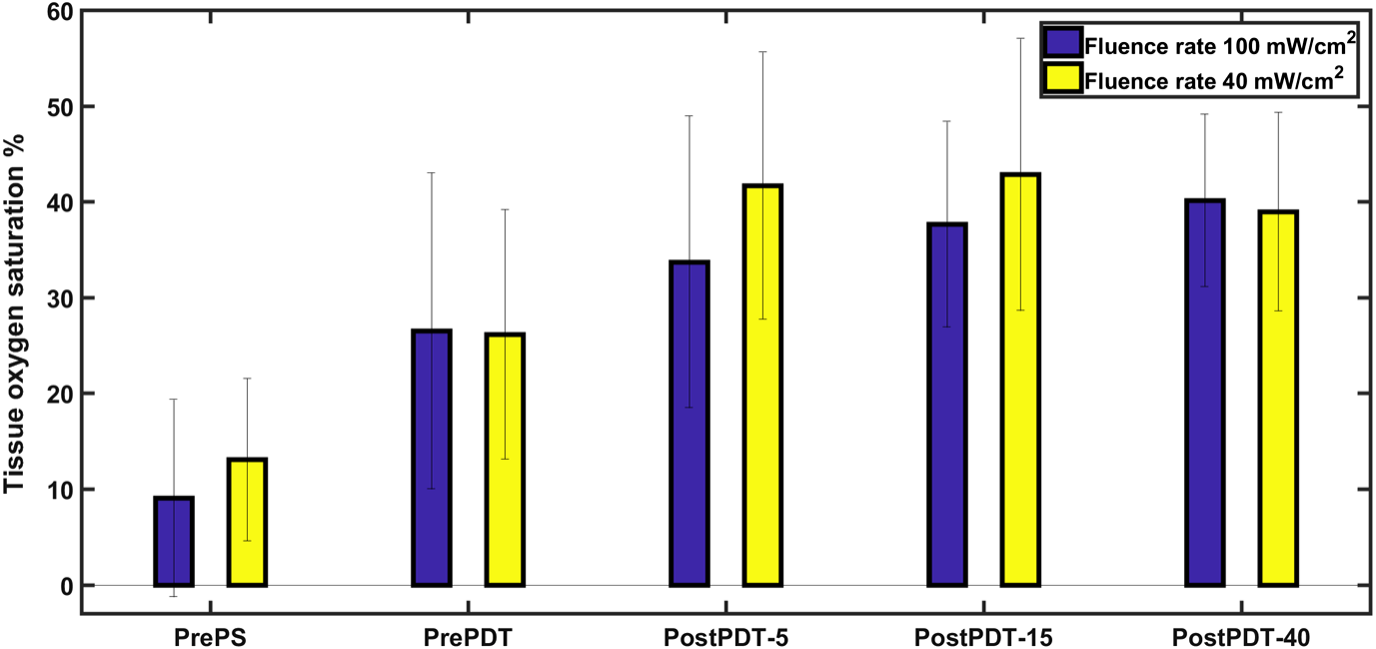
Tumor hemoglobin oxygen saturation measured by the DRS system before drug injection, pre-PDT and post-PDT. PrePS stands for before PS injection, PrePDT for 4 h after injection and before PDT treatment, and PostPDT-5, 15, or 40 for the three different treatment procedures after light delivery. First three box plots on the left represent measurements on 36 animals are divided into two groups of PDT fluence rate (18 animals in each group). The two last box plots show 24 treated animals with $15\;J\;{\mathrm{cm}}^{-2}$ (12 animals in each group) and 12 treated animals with $40\;J\;{\mathrm{cm}}^{-2}$ (6 animals in each group).

In contrast to findings that reveal insignificant optical parameters variation during PDT, blood oxygenation showed a high variation during PDT (Fig. 8). Lowest mean oxygenation was seen before PS injection and it was 9% for group I and 13% for group II. Tissue oxygenation raised to 26% after drug injection and before treatment with p value <0.01 for both groups. The tissue oxygenation increased to 33% (p value <0.01) after treatment using light with $5\;J\;{\mathrm{cm}}^{-2}$ fluence for group I and reached 41% (p value <0.04) for group II. No appreciable difference in PDT effect on oxygen saturation was observed for the rest of the process (nonsignificant p value >0.2). Similar trend is shown in Fig. 5, when the photobleaching rate was constant in the last two stages of treatment.

Histological analysis was planned in order to evaluate tissue changes related to the PDT protocols. However, there were no any significant results from histological analysis likely due to the short time with the sample preparation, which did not allow us to get any relevant information from the slides.

## 4 Discussion

In this study, the change of the important dosimetric parameters for PDT *in vivo*, namely, the tissue optical properties and PS level in tissue were evaluated with a novel combined fluorescence reflectance system with multiemission multiexcitation (MEME) functionality. Measurements of the optical properties of the tissue are of great interest for some medical applications, particularly for PDT as used for dosimetry during the treatment sessions. Dosimetry has the potential to predict treatment outcomes by taking into account nonhomogeneous distribution of PS within the tumor and inter- and intra-animal variability in tissue optical properties. The fiber optic point probe is designed to quantitative tissue fluorescence measurement *in vivo* using separate source–detector fibers. In DRS, short source–detector distance is preferable in order to separate scattering and absorption contribution to the light attenuation. This provides an opportunity to measure with a reasonable good spatial resolution but with the limited penetration depth. The modulation at a kHz frequency regime in this design allows detection of weak fluorescence signals with a variety of illumination sources in the presence of the ambient light. Multi LED sources are employed for fluorescence and reflection spectroscopy. The monitoring of the reflectance is aimed to correct the fluorescence signal from the tissue optical properties and to compensate for source power fluctuation. Different wavelengths will penetrate to different depths. However, in our measurement geometry with a fixed distance between the source and detector fibers, the probe volume is primarily determined by the source–detector distance and only weekly dependent on the wavelength.

Previous studies have shown that the choice of fluence rate in PDT can affect the physiological and cytotoxic response and consequently the treatment outcome.^29^^,^^30^ Therefore, 36 mice received a light dose escalating from $5\;J\;{\mathrm{cm}}^{-2}$ to 15 and $40\;J\;{\mathrm{cm}}^{-2}$ at two fluence rates of 100 and $40\;\mathrm{mW}\;{\mathrm{cm}}^{-2}$. The high fluence rate is chosen based on previous studies when chlorine e6 is used as a PS.^31^ DRS was conducted concurrently to measure tissue optical properties and oxygenation level.

In this study, LS-SVMs were used to evaluate the fluorophore concentration, blood volume, and reduced scattering coefficient. In the phantom calibration experiments, a wide range of absorption and scattering values has been provided to cover a large variation in the optical properties. The PS used in this study was chlorine e6, and it was added to the liquid phantom to validate the accuracy and robustness of the system in the prediction of fluorophore concentration in the presence of large optical variations. The results show that the LS-SVM model can predict the PS concentration in tissues with different optical properties. In addition, the detected signals—blood volume, reduced scattering coefficient, and fluorophore concentration—correlate linearly to the actual concentrations (see Fig. 3). The phantom results were used as a training set in evaluating the same parameters in the *in vivo* measurements. There is a significant variation in optical properties between tumors and within any of the tumors caused by the heterogeneity in tissue. We observed that clear fluctuations are present in blood volume and reduced scattering coefficient in the animals before drug injection prior to the PDT. Such fluctuations would be imperative to consider in PDT treatments, as they indicate that individual tumors can respond differently to PDT.

The intensity of the PS-related signal in tissue was measured with the optical fiber system. The PS level was highly variable among tissues. There is some statistical difference between individual mice, but consistency in average values is clear (Fig. 5). A significant PS uptake is clear after drug administration. A substantial reduction of tissue chlorine e6 after PDT is observed after the first and second treatment due to photobleaching. There are challenges to estimate the PS concentration from fluorescence signals in the presence of endogenous fluorophores. This stems from the background signal from such fluorophores not related to the PS concentration. This could have been avoided by adding these fluorophores in the tissue phantom training set to correct the presence of tissue autofluorescence in tissue.

Our results from both systems show insignificant changes in blood volume and reduced scattering coefficients during the treatment procedure for all light doses and both fluence rates. Such responses have been observed earlier, even though previous studies have investigated other time periods of the post-PDT, possibly explaining differences in the observations.^32^^–^^34^ In most of the studies, the optical property evaluation has been performed for a longer period after PDT, sometimes exceeding 24 months.^32^ These different interrogation times will definitely result in different tumor optical properties, arising from different tumor response stages. For instance, Ahn et al.^32^ presented no appreciable differences in the PDT effect on blood volume as a function of the light dose after PDT, whereas significant changes were reported up to 24 months after treatment.

The hemoglobin oxygen saturation was measured with only DRS to investigate the local tissue oxygenation. In general, most PDT dosimetry studies perform the first oxygenation measurement after drug delivery and before PDT. In this study, we measured the tissue oxygenation before drug injection. Tissue oxygen saturation before PS injection is very low and the probable reason is that the skin tissue can be deoxygenated due to hypoxia. It is expected that skin will contribute significantly to the signal due to the small probe volume interrogated with a short source detection distance. The effect of anesthesia on the oxygen saturation may also be significant for the tissue oxygenation. Interestingly, a slight, yet, significant increase in the oxygenation is observed following drug delivery before any light illumination to the tissue. One explanation could be that after administration of the drug, there might be a physiological response to the drug administration. This trend is also observed after the first treatment stage when tumors were treated with 5 J cm^−2^ light fluence. Understanding the mechanisms of tumor destruction may explain the increase in blood oxygenation after first light delivery. One immediate response to PDT is an acute inflammatory reaction in the targeted site and tumor bed as well as in the normal microvasculature.^11^ An increase in tissue oxygen saturation, while the blood volume is constant, can be explained by increased blood flow (not measured here). Previous reports on blood flow responses due to PDT showed great variability most likely due to differences in tumor characteristics and treatment protocols.^35^^,^^36^ Monitoring of blood flow has been studied with several different techniques, including laser Doppler technique, Doppler optical coherence tomography, and diffuse correlation spectroscopy.^37^^–^^39^ In these studies, an initial increase in relative blood flow velocity followed by a reduction is indicated. Kushan et al. attributed this increase due to the narrowing of the lumen following vasoconstriction. The same trend in vascular response to PDT is observed in a study by Maas et al.^40^ It is also reported that tumor oxygenation change during PDT depends on PS type and time after injection.^41^ Increase in tissue oxygenation was observed for PDT with verteporfin and ALA-induced ALA-PPIX with 3 h between injection and irradiation. The results reported here are encouraging, but more studies are required to translate into clinical use. The significant decrease observed in PS concentration together with a great variation in optical properties can be used to be predictive of response. However, additional interventions may need to be applied to improve the outcome, such as extending the trial time to observe the treatment influence on tumors. Tissue necrosis, apoptosis, and autophagy need longer time to become visible in histological analysis, therefore, further experiments need to be performed with longer evaluation time to get a better understanding of the PDT outcome.

## 5 Conclusions

In conclusion, a fiber-optic probe instrument based on fluorescence and reflectance spectroscopy was used for the purpose of diagnostic and monitoring measurement of tissue. In this study, this system is a complement to PDT as a mean of dosimetry. The PDT treatment outcome depends on many variables, including PS concentration, oxygen saturation, and optical properties variation in tissue. An accurate and reliable dosimetry method to take all these variables into account is essential to allow PDT outcomes to become predictable. In this novel fiber probe system, multiple sources using LEDs enable precise quantification of the fluorophore concentration and tissue optical properties. System performance was validated using LS-SVM regression analysis on phantom with a large variation of optical properties and showed a correlation of >99% between true concentration and predicted value. The quantitative measurement of fluorescence signals was accomplished *in vivo*. Results from this study, together with the results from a previous clinical study,^23^ show encouraging observation for real-time PS quantification during PDT. However, this study was somewhat limited by variation in blood volume concentration, causing changes in absorption and scattering parameters not being significant. Therefore, the future plans include optimization of the system to be utilized for better PDT planning with a more controlled treatment procedure. For example, the longer time interval between irradiation and measurement will be necessary to observe PDT-induced changes in tumors.
